# Mental gravity as a translational framework for mental health promotion

**DOI:** 10.3389/fnhum.2025.1650650

**Published:** 2025-11-25

**Authors:** Lachlan Kent

**Affiliations:** Institute of Health and Wellbeing, Federation University, Melbourne, VIC, Australia

**Keywords:** mental gravity, grounded cognition, anterior insula, mental health promotion, graviceptive imagery, embodiment, emotional regulation, interoception

## 1 Introduction

In recent years, great progress has been made in understanding gravity's impact on cognition (Ferrè et al., 2013), behavior (Gallagher et al., 2019), and health (Spiegel, 2022; Blaber et al., 2010). Based on over a decade's ongoing research into gravity's role in mental health, it may be possible to translate these scientific advances into applied principles for psychological intervention. I am not advocating for gravity-based clinical interventions as such—that would be premature, at best. Instead, I believe there is scope to use gravity-based cognitive science for non-clinical approaches to Mental Health Promotion (MHP).

MHP is a branch of public health and a sub-discipline of health promotion that aims to improve mental health outcomes across populations through empowerment, education, and environmental modification. These interventions complement more individualized, clinical forms of mental healthcare such as psychiatry, counseling, psychotherapy, and psychopharmaceuticals. Rather than focusing on those who are unwell, population-based MHP interventions can take a universal (i.e., everyone), selective (i.e., vulnerable groups), or indicated (i.e., those exhibiting early warning signs) approach to keeping people well (i.e., a preventative approach). Interventions are typically designed to enhance mental health literacy, increase the likelihood of psychological flourishing, and reduce the risk of mental illness (Kent et al., 2025). Traditional MHP models emphasize cognition, emotion regulation, and social determinants of health—all of which are relevant to the cognitive neuroscience of graviception in more or less direct ways, as explained below.

One conceptual framework through which the science could be translated is the novel principle of Mental gravity (MG), which I have proposed as a holistic hypothesis regarding the relation between embodied graviception and mental health (Kent, 2023, 2024). Based in the cognitive neuroscience of simulated graviceptive mental imagery, the MG framework proposes that internal/emotional states are partly organized and interpreted through embodied experiences derived from humans living, developing, and evolving within a gravitational environment. MG seeks to explain how and why mental states are often described in gravitational terms such as “uplifted,” “grounded,” or “off balance,” and why affect (i.e., feelings, emotions, and moods) is especially gravitational in nature (e.g., being depressed, being on edge, feeling of equilibrium, etc.).

Based on these formalized observations, I believe MG could be used as a conceptual framework to meet the three primary aims of MHP: (1) to enhance mental health literacy through embodied spatial metaphors or *vectors* (e.g., upward/stable states are emotionally preferable to downward/unstable states); (2) to increase levels of mental wellbeing by associating upward/outward emotional vectors with positive mental health outcomes (i.e., flourishing); and (3) to reduce the risk of mental illness by associating balance/stability emotional vectors with psychological coping and resilience (i.e., preventing negative mental health outcomes like anxiety and depression). As such, this opinion piece outlines the background and general principles of a future MHP initiative based in MG theory.

## 2 Theoretical foundations

### 2.1 Grounded cognition and embodiment

Grounded cognition theories argue that all cognitive phenomena, including amodal forms (i.e., abstract reasoning or numeric and language processing), are grounded in modal simulations of bodily, affective, perceptual, and motor processes (Barsalou, 2008). This extends embodied cognition to include contextual physical and social factors within which the body is embedded (Pezzulo et al., 2013). Gravity is one such contextual factor. Spatial orientation, force, and verticality are among the most evolutionarily entrenched dimensions of embodiment and environmental embedding (Shapiro, 2019). The cognitive linguistics literature shows strong metaphorical preferences for vertical valence: good is “up,” bad is “down”; control is “centered,” disarray is “off balance” (Cian, 2017; Lakoff and Johnson, 2008).

Grounded cognition has implications for mental health and wellbeing. For example, negative emotions are felt as bodily heaviness, while positive emotions are felt as lightness (Hartmann et al., 2022). Furthermore, gravity influences emotion-related physiology such as gut health (e.g., visceral anxiety along the gut-brain axis) and serotonergic neurotransmission (Spiegel, 2022, 2025). Gravity also influences social and emotional cognition by, for example, contributing to cognitive aspects of selfhood (Ferrè et al., 2014), bodily awareness (Ferrè et al., 2013), and ego- vs. allocentric perspective-taking (Pavlidou et al., 2018). To summarize these effects, MG hypothesizes that gravity shapes emotional experience in global, non-specific ways including, importantly, experiences of mental health and ill-health in conditions like depression (i.e., the “fallen” emotional state) and anxiety (i.e., the “falling” emotional state; Kent, 2024).

Depression serves as the canonical example of MG where people tend to feel down, low, heavy and slow “as though” their mind and body are laboring under an excessive, overwhelming downward pull of gravity (Kent, 2023). The “as though” is crucial here because it refers to the modal (concrete or bodily) simulations of amodal (abstract or dis-embodied) information referred to above in relation to grounded cognition. The depressed person experiences an abstract emotional state via simulated mental imagery as an embodied “down” vector in socio-emotional space.

### 2.2 Graviception and neural architecture

The anterior insula cortex has been identified as a convergence zone for modal bodily representations of interoception, emotional awareness, and vestibular (graviceptive) information (Gu et al., 2013; Uddin et al., 2017). The salience network, in which the anterior insula plays a central role, helps detect and respond to emotionally and physically relevant stimuli by gating this modal information between the central executive network (i.e., externally-oriented information) and default mode network (i.e., self-oriented information; Goulden et al., 2014; Molnar-Szakacs and Uddin, 2022).

The anterior insula has been centrally-implicated in the development of common mental health conditions like depression (Manoliu et al., 2014; Palacek et al., 2025) and anxiety (Geng et al., 2016; Li et al., 2023), as well as less common conditions like schizophrenia (Menon et al., 2023; Nelson et al., 2014). The anterior insula and salience network have also been linked to core facets of subjective wellbeing (Lewis et al., 2014; King, 2019).

Crucially for MG, in addition to these general functions and sequelae, the anterior insula is also responsible for simulating bodily orientation during imagined, fictive movement (i.e., without sensory feedback), in contrast to the posterior insula cortex which integrates sensory feedback during non-fictive, physical movement (Rousseau et al., 2020). Given the anterior insula's dual role in simulated graviception and emotional awareness, it represents the cognitively-grounded neural correlate/mechanism underpinning MG via the integration of homeostatic, affective, and spatial inputs (Kent, 2024).

### 2.3 Mental gravity model

MG further proposes that these gravitational simulations create vectors in mental space—or more accurately, cognitive spacetime (Kent, 2024; Stocker, 2014). This means that they can apply to abstract social and emotional concepts like the developmental trajectories of selfhood, autobiographical narrative, and value (Kent, 2024). The anterior insula co-opts these amodal vectors to structure deep interoceptive inference regarding affective/social cognition and representations of homeostatic/allostatic balance (Seth and Friston, 2016). In other words, the anterior insula maps spatial vectors onto affective information to yield emotional valence:

- Inward: positive valence = emotional centredness (i.e., focus, awareness, and containment), negative valence = emotional diffusion (i.e., lack of focus, awareness, or containment).
- Downward: positive valence = emotional stability (i.e., grounded, calm, and controlled), negative valence = emotional instability (i.e., ungrounded, volatile, or uncontrolled).
- Upward: positive valence = emotional elevation (i.e., pleasant, enjoyable, and desirable), negative valence = emotional lowness (i.e., unpleasant, unenjoyable, or undesirable).
- Outward: positive valence = emotional openness (i.e., connected, expressive, and receptive), negative valence = emotional closedness (i.e., disconnected, suppressive, or unaccepting).

I propose that these vectors can be translated into practical MHP tools to increase understanding of emotional dynamics (i.e., mental health literacy), increase positive emotionality (i.e., flourishing), and decrease negative emotionality (i.e., distress or mental illness).

## 3 Mental gravity in health promotion

Spatial vectors are powerful language devices that people use in everyday language and metaphor to understand and communicate their emotional experience (Cian, 2017; Lakoff and Johnson, 2008; Stocker, 2014; Coll-Florit et al., 2021). Gravitational language can be used formally to communicate key MHP concepts, while also suggesting new ways to frame common health-promoting activities such as physical activity (i.e., upward vectors), grounding exercises (i.e., downward vectors), mindfulness practices (i.e., inward vectors), and pro-social attitudes (i.e., outward vectors).

These practices are well-supported in the intervention literature. Systematic reviews demonstrate that mindfulness meditation enhances interoception and reduces distress (Sharp et al., 2018), yoga improves self-regulation and resilience (Gard et al., 2014; Schillings et al., 2021), and nature immersion is linked to wellbeing gains across large population samples (White et al., 2019). Posture, gait, and balance training also show measurable preventive effects on depression and anxiety (Canales et al., 2017; Feldman et al., 2020).

These interventions are typically evaluated with respect to whether they: (a) increase mental health literacy (i.e., knowledge and attitudes about psychological health and wellbeing); (b) increase the capacity of the individual to experience positive thoughts, feelings and behaviors (i.e., hedonic or eudaimonic wellbeing in the form of positive emotion, life satisfaction, or quality of life); or (c) reduce the likelihood that an individual will experience negative thoughts, feelings and behaviors (i.e., psychological distress, psychosocial impairment, and/or a mental health condition like depression and anxiety).

In terms of the latter two aims of MHP, one of the key intervention targets is emotional regulation (Menefee et al., 2022), which involves either up-regulating positive emotions to improve wellbeing (Livingstone and Srivastava, 2012) or down-regulating negative emotions to reduce distress or psychosocial dysfunction, such as in response to trauma (Xion et al., 2013). Mindfulness improves emotional regulation through initial down-regulatory interventions such as non-judgemental awareness and acceptance of feelings. Nature immersion, on the other hand, can upregulate positive feelings of awe, connection and immersion.

The MG paradigm can interpret these different modes of MHP promotion/up-regulation and prevention/down-regulation as emotional vectors and embodied gravitational states of balance/stability or elevation/lightness. This can, in turn, improve mental health literacy by providing a concrete, physical, and intuitive framework for consumers to understand the mechanics of MHP.

From this perspective, the MG paradigm can support the three pillars of MHP at the individual level of intervention:

- Mental health literacy: enhancing understanding of spatial-emotional metaphors, embodied cognition, and cognitive spacetime; linked to improved attitudes toward mental health and earlier intervention (Johnson et al., 2023).
- Promotion of mental wellbeing: using upward and outward simulation vectors to evoke positive emotions, self-efficacy, and exploration of physical and social environments; linked to states of flourishing as opposed to languishing (Iasiello et al., 2024).
- Prevention of mental ill-health: using downward and inward simulation vectors to promote emotional stability and psychophysiological (autonomic) balance; linked with reduced incidence of mental health conditions such as anxiety and depression (García-Campayo et al., 2015; Bellón et al., 2015).

Prevention in mental health occurs at many levels from individual to dyads to family units, and from educational and occupational environments to wider spheres of community and society. Addressing social determinants is imperative but crucial individual- and family-level interventions typically aim to increasing resilience to adversity, trauma and other stressors through improved social and emotional competence (Arango et al., 2018). Using concrete, embodied concepts of emotion's physical characteristics can aid competence through understanding, especially for young children (Hoemann et al., 2019) who stand to benefit from targeted mental health literacy interventions (Tully et al., 2019).

These aims can be achieved through psychological and educational programs designed to improve self-care, create sustained individual behavior change, and increase resilience through stress management. Interventions could be applied to general audiences and target populations as needed, including youth and even young children given the intuitive nature of emotional vectors/metaphors. Some example interventions are detailed below.

### 3.1 Graviceptive imagery practices

A MG-MHP protocol can include specific practices to target graviceptive simulation:

- Mindfulness meditation: often seated and still, reinforces inward focus; shown to activate anterior insula and enhance interoception (Sharp et al., 2018).
- Floatation therapy: induces buoyancy and simulated weightlessness; linked to interoceptive awareness (Pantazis and Wittmann, 2025) and reduced symptoms of depression and anxiety (Garland et al., 2024).
- Vertical gaze exercises: engage upward simulation to support emotional positivity; linked to vestibular alignment (Meldrum and Jahn, 2019), aesthetic appreciation (Gallagher and Ferrè, 2018), and depression (Mańkowska et al., 2020).

### 3.2 Environmental engagement practices

A MG-MHP protocol includes specific practices targeting more active engagement with the gravitational environment:

- Nature immersion: wide horizons and natural terrain support openness, awe, and subjective wellbeing (White et al., 2019).
- Movement routines: walking, yoga, and slow movement practices reinforce gravitational interaction; linked to interoceptive accuracy, self-regulation and psychological health (Gard et al., 2014; Schillings et al., 2021).
- Gait, posture and balance exercises: physical activity to improve core, spinal, and whole-body alignment through dynamic postural control; linked to symptoms of depression and anxiety (Canales et al., 2017; Feldman et al., 2020).

### 3.3 Biopsychosocial practices

Other interventions could be designed to target physical and social health as indirect pathways to psychological wellbeing.

- Gut-brain axis: dietary and other gut-health interventions that influence emotional experience via the gravitationally-mediated gut-brain axis (Spiegel, 2022); linked to depression risk (Lassale et al., 2019) and other indices of positive and negative affect (Lee et al., 2020).
- Serotonergic signaling: considered as a global mediator of gravity management (Spiegel, 2025), interventions to increase synthesis/availability of gut-derived serotonin may yield mental health benefits associated with enhanced body-brain graviceptive simulations; linked to positive and negative mental health outcomes (Nestor et al., 2021; Arnone et al., 2024).
- Virtual reality (VR): similar to altered emotional vectors induced by psychedelic therapy (i.e., spatial and temporal distortions from serotonergic drugs like psilocybin), group VR exercises can induce upward/outward emotional vectors in the form of self-transcendent experiences; linked with coping flexibility, stress reduction and improved mental wellbeing (Arslan et al., 2025).

## 4 Discussion

The MG-MHP framework suggests that improving emotional wellbeing and resilience are not only about thoughts and feelings, but also about reengaging embodied cognition. By anchoring emotional regulation in graviceptive imagery and vector simulation, MG-MHP offers a neuroscientifically- and cognitively-grounded approach to mental health and wellbeing. The anterior insula emerges as both a neural gateway and a target of MG-MHP interventions.

MG can add unique value to established MHP frameworks. The three pillars—literacy, wellbeing, and prevention—map naturally onto MG's vectors. Literacy is supported by embodied metaphors that clarify emotional states; wellbeing is enhanced by upward and outward practices linked to flourishing; prevention is strengthened by inward and downward practices that promote stability and resilience. MG also complements resilience training by offering an embodied scaffold for coping strategies, and aligns with positive psychology by reframing flourishing as gravitational elevation and openness.

For example, MG-MHP could translate the “broaden-and-build” theory of positive emotion (Huppert et al., 2004), which falls under the positive psychology umbrella, through the use of vector-based embodied cognition practices. This would: (a) increase wellbeing literacy though improved understanding of what comprises positive emotion; (b) enhance wellbeing by linking bodily sensations to positive emotions; and (c) strengthen resilience by empowering individuals to identify and respond to the absence of positive emotion (i.e., a risk-factor for negative emotion, distress, and anxiety/depression).

MG-MHP provides a new foundation for mental health promotion rooted in the body's relationship to gravity. Through embodied simulation of inward, downward, upward, and outward emotional vector-states, individuals may achieve greater understanding of the internal and external forces shaping their mental health, helping individuals and populations become more likely to flourish and less likely to experience mental ill-health. There is further scope to scale MG-MHP concepts up to the collective level of social determinants of mental health using analogous societal vectors.
